# Primary care doctors in acute call-outs to severe trauma incidents in Norway – variations by rural-urban settings and time factors

**DOI:** 10.1186/s12873-024-01027-5

**Published:** 2024-06-26

**Authors:** Kristian Rikstad Myklevoll, Erik Zakariassen, Tone Morken, Valborg Baste, Jesper Blinkenberg, Gunnar Tschudi Bondevik

**Affiliations:** 1 Section for General Practice, Department of Global Public Health and Primary Care, University of Bergen, Postbox 7804, Bergen, N-5020 Norway; 2 National Centre for Emergency Primary Health Care, NORCE Norwegian Research Centre, Postbox 22 Nygårdstangen, Bergen, N-5838 Norway

**Keywords:** Advanced trauma life support care, Emergency medical services, Norway, Out-of-hours medical care, Primary health care, Rural health service, Triage

## Abstract

**Background:**

A severely injured patient needs fast transportation to a hospital that can provide definitive care. In Norway, approximately 20% of the population live in rural areas. Primary care doctors (PCDs) play an important role in prehospital trauma care. The aim of this study was to investigate how variations in PCD call-outs to severe trauma incidents in Norway were associated with rural-urban settings and time factors.

**Methods:**

In this study on severe trauma patients admitted to Norwegian hospitals from 2012 to 2018, we linked data from four official Norwegian registries. Through this, we investigated the call-out responses of PCDs to severe trauma incidents. In multivariable log-binomial regression models, we investigated whether factors related to rural-urban settings and time factors were associated with PCD call-outs.

**Results:**

There was a significantly higher probability of PCD call-outs to severe trauma incidents in the municipalities in the four most rural centrality categories compared to the most urban category. The largest difference in adjusted relative risk (95% confidence interval (CI)) was 2.08 (1.27–3.41) for centrality category four. PCDs had a significantly higher proportion of call-outs in the Western (RR = 1.46 (1.23–1.73)) and Central Norway (RR = 1.30 (1.08–1.58)) Regional Health Authority areas compared to in the South-Eastern area. We observed a large variation (0.47 to 4.71) in call-out rates to severe trauma incidents per 100,000 inhabitants per year across the 16 Emergency Medical Communication Centre areas in Norway.

**Conclusions:**

Centrality affects the proportion of PCD call-outs to severe trauma incidents, and call-out rates were higher in rural than in urban areas. We found no significant difference in call-out rates according to time factors. Possible consequences of these findings should be further investigated.

## Background

Norway has 5.4 million inhabitants, with 80% of the population living in urban areas, mainly along the coastline in Southern Norway [1]. Inhabitants in rural areas may have long distances to health care services.

Most of the research on prehospital trauma care in rural areas with long distances to trauma hospitals is from the USA, Canada and Australia [2–4]. The research has documented both higher mortality rates and the benefits of a locally adapted inclusive trauma system in rural areas. Norway is a high-income country with publicly funded health care and a national trauma care system. The trauma care system is similar to what is found in the other Nordic countries, which have comparable transport distances, climate, political systems and demographics [5].

There have been two main systems of prehospital trauma care worldwide. One system is based on acute care performed by paramedics and a “load-and-go” strategy from the incident scene, where an ambulance takes the patient to the emergency department at a local trauma hospital [6–8]. The second system brings the physician to the incident scene and gives the opportunity to assess the patient there. In several countries, the physicians involved in prehospital trauma care are usually specialists in emergency medicine or anaesthesiology.

Unlike the other Nordic countries, Norway and Iceland use a variant of the second system were the primary care doctor (PCD) may call out to severe trauma incidents as the physician in a team together with the ambulance services. PCDs and the ambulance services have been described as the backbone of the prehospital emergency care in Norway [9]. They may, however, be supported by an anaesthesiologist from the Helicopter Emergency Medical Services (HEMS).

A severely injured patient needs treatment according to the trauma chain of survival, which includes early first aid, prehospital treatment, advanced treatment at a trauma hospital and rehabilitation [8–11]. Fast transportation to a trauma hospital for definitive care should be given high priority.

In Norway, the provision of prehospital emergency services is a shared responsibility between the primary and secondary healthcare systems. The municipalities hold the responsibility for emergency primary health care. The latest regulation from 2015 underlines the requirement to have PCDs on call 24 h a day, emergency primary care centres and acute health care provision for all people in their districts. On-call PCDs must be able to call out immediately when required. Many emergency primary care centres therefore have a rapid response vehicle available [12]. Previously, PCDs in many municipalities called out together with the ambulance or by taxi, with a risk of delaying the response. By January 2022, there were 168 emergency primary care centres in Norway [13]. In 2022, 60% of these centres had their own rapid response vehicle for PCD call-out. This figure had increased from 36% in 2014, and 19% in 2009 [13].

Secondary health care is organised by the national Government through four Regional Health Authority areas (South-Eastern Norway (South-East), Western Norway (West), Central Norway (Central), Northern Norway (North)), which are responsible for the hospitals, the ambulance services, the HEMS and the Emergency Medical Communication Centres (EMCCs) in their area. By 2021, the secondary health care services operated 520 ambulances and 14 HEMS [14, 15]. Norway is divided into 16 EMCC areas. Each EMCC is responsible for emergency medical communication in their local area.

Norway has four level-one trauma centres, one for each health trust, in addition to 37 district trauma hospitals. A level-one trauma centre provides trauma care for all injuries and is obliged to have available all relevant specialities and support functions, trauma meetings and trauma registers [10, 11].

Norwegian district trauma hospitals are level-two or level-three trauma centres. They are obliged to have a trauma team available around the clock, and there are requirements for competence and joint training. These hospitals should be able to treat the majority of injured patients. If the most severely injured patients are not transported directly to a level-one trauma centre, the district trauma hospitals must provide the correct initial treatment and have routines for transfer to level-one trauma centres when needed.

If anyone experiences a life-threatening injury, they should call the national emergency number 113 to the EMCC [12]. If telephone triage concludes with a potentially life-threatening situation, it will be classified as an “acute response” incident. In accordance with national guidelines, the EMCC should send an alarm to the on-call PCD and the ambulance services. The alarm is also sent to the HEMS, if necessary. With the alarm and primary message, the EMCC dispatches ambulances. The on-call PCDs must decide their response. They could stay at the emergency primary care centre or call out to the incident scene.

Although there are national guidelines for prehospital trauma care [10], studies have shown variations regarding PCD involvement and call-outs [9, 16]. In a previous study, we found that PCDs called out to only 38% of the severe trauma incidents of which they had been notified [16]. Increased proportions of PCD call-outs to severe trauma incidents were significantly associated with lower age of the PCD, being a GP specialist, lower patient age, and male patient gender.

The frequency of trauma incidents may vary according to season, day of the week and time of day. Consequently, the emergency health services should be strategically planned and staffed, to guarantee sufficient resources at all times, throughout the year, week and day. There might be variations in PCDs threshold for call-outs and potential delays during specific periods, such as in the middle of the night, the winter season, or during peak hours at the emergency primary care centres.

The present study aimed to investigate how variations in rural-urban settings and time factors were associated with PCD call-outs to severe trauma incidents in Norway. The findings may affect the local organization of health services, including ensuring adequate staffing levels, providing support to the PCDs, enhancing competence development in emergency primary care, organizing duty schedules, and equipping emergency primary care centres with necessary tools and equipment for call-outs to severe trauma incidents.

## Methods

This study was based on registry data obtained for all acute admissions to Norwegian hospitals in the seven-year period 2012–2018 from the Control and Payment of Reimbursement to Health Service Providers database (KUHR), the Norwegian Patient Registry (NPR), the National Out-Of-Hours Services Registry (NOOHR) and Statistics Norway. The methods have been described in a previously published article [16]. In the present study, a PCD was defined as an on-call doctor, working either as a general practitioner (GP) or an out-of-hours physician.

Norwegian PCDs submit a claim to the KUHR after each patient contact. These claims contain patient information, including the patient’s national identification number, the contact date and time, and the type of contact (clinic consultation, telephone contact or home visit/call-out). The NPR includes information about the patient’s national identification number, the date and time of admission, and the degree of urgency. The physician at the hospital uses an International Statistical Classification of Diseases and Related Health Problems Version 10 (ICD-10) code to register the patient’s diagnosis. An injury is registered as Block S-T, or Chap. 19, which refers to diseases classified as Injuries, Poisoning and Certain Other Consequences of External Causes. The NPR also contains Diagnosis-Related Group (DRG) codes made by the hospital. The DRG code is made by the ICD-10 code and other patient information. The DRG code reflects the hospital resource used during the hospital stay and is used by the Government to finance the hospitals.

To calculate the distance to the nearest hospital, we found the addresses of the emergency primary care centres from the NOOHR. For the years 2016 and 2018, the NOOHR had data on which of the emergency primary care centres had a rapid response vehicle available for immediate call-out. Statistics Norway replaced the personal identification number with a pseudo-anonymised identification number in the NPR and the KUHR, which made it possible to link data from both registries without revealing patients’ identities.

The research datasets were produced by linking acute admissions to hospitals in the NPR database with a previous PCD contact in the KUHR database. We assumed that a PCD contact less than 24 h before a hospital admission due to severe trauma was a PCD involvement in the same incident. Hence, we linked PCD contacts in the KUHR and the corresponding hospital admissions in the NPR. A previous study validated this algorithm to identify PCD referral contacts for acute hospital admissions between acute hospital admissions and PCD consultations less than 24 h before the admission [17, 18].

The PCDs complete the compensation claims to KUHR when they have time. Therefore, some of the claims in our study were completed after the admission. All these patients were admitted for at least 24 h, and it is likely that the PCDs had carried out the patient treatment before the admission. The time on the KUHR account card is registered automatically and will not change back to the time of the trauma incident. We therefore included PCD consultations documented either less than 24 h before or less than 12 h after admission.

We included all patients discharged from all hospitals in Norway during the period 2012–2018 with some selected severe traumas as their main diagnosis, whereby a PCD call-out response could be expected. The selected severe trauma DRG codes were Craniotomy at significant multi-trauma, Major surgery hip/femur and replantation, Operations at significant multi-trauma, and Significant multi-trauma (DRG codes 484–487). It is likely to presume that these instances of severe trauma prompted acute alarms to the on-call PCDs and ambulance services.

Statistics Norway uses centrality as one of the criteria in the classification of municipalities. Each municipality is given a value reflecting its degree of centrality and is further assigned to one of six centrality groups. Centrality group one is the most urban, and group six is the most rural. Each group consists of municipalities from all parts of Norway with the same degree of centrality, except centrality group one, which includes six municipalities bordering the capital.

We recorded three geographic variables indicating rural-urban settings: centrality group defined by Statistics Norway (centrality groups 1–6); Regional Health Authority area (South-East, West, Central, North); and distance from the emergency primary care centre to the hospital (< 1 km, 1–30 km, > 30 km). Further, we recorded three time-factor variables: time of year in three-month periods (December–February, March–May, June–August, September–November); day of week (weekday or weekend – weekends did not include holidays); and time of day (08–16, 16–24, 00–08). We also recorded PCD age, GP speciality, patient age and patient gender. Finally, we recorded whether the emergency primary care centre had a rapid response vehicle for immediate PCD call-out (available data for the years 2016 and 2018).

As we did not, have access to information directly from the EMCCs, we allocated the included patients to the EMCC areas according to the information from the NPR. We assigned the incidents to their specific local EMCC areas based on the patients’ home municipalities and used these incidents to calculate the frequencies of PCD call-outs for each specific EMCC area. Finally, we used the number of inhabitants to calculate the rate and the confidence intervals of PCD call-outs per 100,000 inhabitants per year across the 16 EMCC areas. To study the associations between rural-urban and time characteristics and the likelihood of PCD call-out to severe trauma incidents, generalised linear models (GLM) were used to estimate relative risks (RR) with 95% confidence intervals (CI). Both crude and adjusted models were provided. In a previous study, lower PCD age, being a GP specialist, lower patient age and being a male patient were factors that significantly increased the PCD call-out rate [16]. We performed sensitivity analyses by including each of the four variables in the adjusted regression model, one by one. Both PCD age and GP speciality affected the results significantly and were included in the final adjusted model in addition to the three geographic and three time variables.

Based on data from 2016 to 2018, GLM was used to estimate the association between the availability of a rapid response vehicle and the likelihood of PCD call-out. Both crude and adjusted (for rural-urban and time characteristics, PCD age and GP speciality) estimates are presented. Finally, to further illustrate the variation, we calculated the call-out rates per 100,000 inhabitants per year for the selected severe traumas across the 16 EMCC areas in Norway.

Statistical significance was set at α = 0.05, and the data was analysed by the statistical program Stata 16.1. (StataCorp. 2019. Stata Statistical Software: Release 16. College Station, TX: StataCorp LLC.).

## Results

During the period from 2012 to 2018, the number of acute hospital admissions in Norway were 3,864,433. Among these admissions, 520,098 were related to injuries categorized with ICD-10 codes within Block S-T. There were 4,342 acute admissions specifically linked to the four chosen severe trauma DRG codes. PCDs were involved in 1,683 (39%) of these trauma incidents. There was no documented PCD involvement in the remaining 2,659 incidents (61%) that were admitted to the hospitals. Of the 1,683 severe trauma incidents that involved PCDs, they called out 644 (38%) times.

Compared to the most urban centrality group (group one), there were significantly higher proportions of PCD call-outs to severe trauma incidents in municipalities in centrality groups three, four, five and six, with RR (95% CI) 1.70 (1.10–2.31), 1.97 (1.27–3.07), 1.73 (1.09–2.76) and 2.08 (1.27–3.41), respectively (Table 1). In the Regional Health Authority areas West and Central, PCDs had significantly higher proportions of call-outs compared to PCDs in the most urban, South-East area, with RR 1.46 (1.23–1.73) and 1.30 (1.08–1.58), respectively.

**Table 1:** Primary care doctor (PCD) call-outs to severe trauma incidents, rural-urban and time factors, Norway, 2012–2018

Variable											
	Severe trauma	Primary care doctor (PCD) call-outs	%	RR_crude_^1^		95% Confidents interval (CI)	RR_ad_^2^	95% CI^3^			
**Total** ^4^	1,683	644	38.3								
**Centrality group**											
1 (most urban)	143	26	18.2	1.00			1.00				
2	293	98	33.5	1.84	1,25 − 2.70		1.48	0.94–2.31			
3	445	160	36.0	1.98	1.37–2.86		1.70	1.10–2.63			
4	454	210	46.3	2.54	1.77–3.65		1.97	1.27–3.07			
5	242	101	41.7	2.30	1.57–3.35		1.73	1.09–2.76			
6 (most rural)	92	45	48.9	2.69	1.79–4.04		2.08	1.27–3.41			
**Regional Health Authority area**											
South-Eastern	731	208	28.5	1.00			1.00				
Western	441	213	48.3	1.70	1.46–1.97		1.46	1.23–1.73			
Central	322	140	43.5	1.53	1.29–1.81		1.30	1.08–1.58			
Northern	184	82	44.6	1.57	1.28–1.91		1.18	0.93–1.50			
**Distance from emergency primary care centre to hospital**											
< 1 km	653	241	36.9	1.00			1.00				
1–30 km	543	187	34.4	0.93	0.80–1.09		1.01	0.85–1.19			
> 30 km	482	215	44.6	1.21	1.05–1.39		1.09	0.94–1.28			
**Month**											
Dec – Feb	349	116	33.2	1.00			1.00				
Mar – May	423	168	39.7	1.19	0.99–1.44		1.12	0.92–1.36			
Jun – Aug	466	195	41.9	1.26	1.05–1.51		1.11	0.92–1.34			
Sep – Nov	445	165	37.1	1.12	0.92–1.35		1.04	0.85–1.27			
**Day**											
Weekday	1,204	452	37.5	1.00			1.00				
Weekend excl holidays	479	192	40.1	1.07	0.94–1.22		1.08	0.94–1.25			
**Time**											
08–16	712	270	37.9	1.00			1.00				
16–24	756	311	41.1	1.08	0.96–1.23		1.14	0.99–1.30			
00–08	210	62	29.5	0.78	0.62–0.98		0.99	0.76–1.29			

^1^Crude relative risk (RR_crude_) obtained from generalized linear model (GLM)

^2^Adjusted relative risk (RR_adj_) obtained from GLM adjusted for all the variables included in this table, and the variables PCD age and GP specialty

^3^Confidence interval ^4^Some of the variables have less observations due to missing data. The variable centrality group has 1669 included incidents and 640 call-outs

The variables Regional Health Authority area, distance to hospital and time have 1678 incidents and 643 call-outs. The other variables have 1683 incidents and 644 call outs

There were no significant differences in the proportions of PCD call-outs to severe trauma incidents when comparing emergency primary care centres with or without a rapid response vehicle, RR (95% CI) 0.89 (0.71–1.11) (Table 2).

**Table 2:** Primary care doctor (PCD) call-outs to severe trauma incidents according to rapid response vehicle, Norway

	All incidents involving PCDs^1^	Call-outs					
Available rapid response vehicle	N	n	%	RR_crude_	95% CI^2^	RR_adj_^3^	95% CI
No	265	104	39.3	1		1	
Yes	269	94	34.9	0.89	0.71–1.11	0.89	0.71–1.11

^1^Avaliable data for the years 2016 and 2018

^2^Confidence interval

^3^Adjusted relative risk (RR) obtained from generalized linear model adjusted for the variables centrality group, Regional Health Authority area, distance from the emergency primary care centre to the hospital, time of year, day of week, time of day, PCD age and GP speciality

We were able to identify the corresponding EMCC in 1,677 of the 1,683 included severe trauma incidents. The PCD call-out rates ranged from 17 to 55% across the EMCCs, with an average of 38% (Table 3). PCD call-outs per 100,000 inhabitants per year ranged from 0.47 (0.13–0.80) to 4.71 (2.25–7.16), with a mean call-out rate of 1.81 (1.44–2.19).

**Table 3:** Primary care doctor (PCD) call-outs to severe trauma incidents, Emergency Medical Communication Centres, Norway, 2012–2018

EMCC^1^	Primary care doctor (PCD) call-out to severe trauma incidents, 2012-18					
	PCD involved	PCD call-outs		Inhabitants	Call-out rate per 100,000 inhabitants per year	
	N^2^	n	%	N	rate	95% CI^3^
Total	1,677	642	38.3	5,055,195	1.81	1.44–2.19
Finnmark	31	17	54.8	77,620	3.13	-0.81-7.06
Sør-Trøndelag	99	51	51.5	324,289	2.25	0.62–3.88
Haugesund	90	46	51.1	178,571	3.68	0.87–6.49
Sørlandet	197	99	50.3	300,396	4.71	2.25–7.16
Bergen	148	74	50.0	442,742	2.39	0.95–3.83
Stavanger	142	68	47.9	360,376	2.70	1.00-4.39
Bodø	40	19	47.5	139,110	1.95	-0.37-4.27
Nord-Trøndelag	89	40	44.9	147,994	3.86	0.70–7.03
Tromsø	82	34	41.5	190,625	2.55	0.28–4.81
Førde	57	22	38.6	111,185	2.83	-0.30-5.95
Møre & Romsdal	136	51	37.5	257,720	2.83	0.77–4.88
Helgeland	29	10	34.5	77,010	1.86	-1.19-4.90
Vestre Viken	96	23	24.0	281,336	1.17	-0.09-2.43
Oslo	225	51	22.7	1,563,669	0.47	0.13–0.80
Vestfold Telemark	113	20	17.7	265,057	1.08	-0.17-2.33
Innlandet	103	17	16.5	337,495	0.72	-0.19-1.62

^1^Emergency medical communication centre (EMCC)

^2^Corresponding EMCC identified in 1,677 out of the 1,683 included severe trauma incidents and 642 out of the 644 call-outs

^3^Confidence interval

## Discussion

The proportion of PCD call-outs to severe trauma incidents was higher in rural than in urban areas. This was shown directly in the variable centrality group, but also indirectly in the variables regional Health Authority area and EMCC area. Compared to the most urban (centrality group one), there were significantly higher proportions of PCD call-outs to severe trauma incidents in more rural municipalities (centrality groups three, four, five and six). In the regional Health Authority areas West and Central, PCDs had significantly higher proportions of call-outs compared to PCDs in the most urban area South-East. We observed a large variation both in proportions of PCD call-outs to severe trauma incidents and call-out rates per 100,000 inhabitants per year across the 16 EMCC areas.

Several studies comparing prehospital trauma care in urban and rural areas have found higher mortality rates after trauma, fewer emergency medical health personnel per inhabitant, and longer distances from the incident scene to the trauma hospital in rural areas [6–8, 19–25]. Some studies also observed that injuries were more severe in rural than in urban areas [19, 20, 22]. It is likely that the same challenges apply to rural areas in Norway. This study is unique as it analysed factors affecting PCD call-outs to severe trauma based on national data. This provided insight into the overall frequency of call-outs as well as rural-urban differences.

Due to fewer emergency medical health personnel and longer transport distances to trauma hospitals requiring continuous assessment and urgent measures, PCDs in rural areas have a more important role in prehospital trauma care compared to urban areas [12, 26]. PCDs working in rural areas are aware of the limited availability of medical resources, including ambulances and the HEMS. This may partly explain why PCDs in rural areas tend to call out to incident scenes more frequently than PCDs in urban areas. Further, the workloads are often less intense than in urban emergency primary care centres, which makes it easier for the rural PCDs to call out.

In rural areas, the PCDs work in the same local community as they live in, whilst urban GPs often only meet their patients in the course of their work. Due to relations to the local population, rural PCDs may have a stronger sense of loyalty to colleagues, patients and their relatives, and this may lower the threshold for them to call out to trauma incidents [26, 27]. As rural PCDs often know the patient population, a personal assessment with high-context communication at the incident scene may give better patient care. This could be an additional motivation for the PCDs to call out [28].

Severe trauma incidents are relatively rare in rural areas. When such an incident happens, it is common for the inhabitants of the community to be interested in the incident and discuss it afterwards. They may be interested in which personnel from the emergency services attended, including the PCD. This may lead to increased expectations of the PCDs regarding the right competence and participation in emergency care in the event of incidents in the local community [29].

Due to the higher number of patient contacts, Norwegian ambulance personnel working in urban areas are more experienced than those working in rural areas. Similar rural-urban differences have been reported in studies from other countries [30, 31]. In larger cities, there may be dedicated ambulances with anaesthetists who can call out to incidents with severe trauma [15]. With multiple health care resources available and short transport time and distance to the nearest trauma hospital, there may be less need for PCDs to call out to the incident scenes in urban areas.

In the present study, proportions of PCD call-outs to severe trauma incidents were significantly lower in the most urban regional Health Authority area compared to in two of the other regional Health Authority areas. This has not previously been investigated across all regional Health Authority areas in Norway within a single research study.

A Norwegian study from 2010 found that the GP call-out proportion when alerted by an emergency medical alarm from the EMCC ranged from 40 to 47%. The material was collected from three EMCCs in two different regional Health Authority areas [32]. These findings are comparable with three of the four Regional Health Authority areas in our study. Norway’s four regional Health Authority areas have variations in demographics. In the present study, we have adjusted for centrality and distance, and could argue that the observed differences between the four health trusts could also be related to variations in the organisation and planning of the prehospital trauma care. The PCDs are largely influenced by decisions made by the specialist health care system, even though they work in the primary health care system. The Regulation on the organisation of emergency services states that the municipalities and the Health Authority areas must ensure that the population in need of immediate help receives appropriate and coordinated emergency medical services outside hospitals [12].

Interestingly, we did not observe any significant differences in the proportions of PCD call-outs to severe trauma incidents according to time factors. In the adjusted analyses, neither time of the year, weekday or time of the day were significantly associated with PCD call-out. An American study observed no delay in acute trauma operations for patients admitted at night [33]. A well-functioning trauma system was suggested as the likely cause. A German study reported a slightly increased prehospital time delay for patients admitted at night, although there was no documented clinical impact. Increased prehospital time was related to time-consuming procedures and lower staffing levels at night [34]. PCDs could be expected to call out less frequently in the winter season when the weather is bad. Three different studies found differences in trauma incidents across seasons, but no differences in mortality [35–37]. The same studies observed that the use of health resources in trauma incidents in hospitals varied across seasons.

We found no significant difference in call-out rates across emergency primary care centres with or without rapid response vehicles. As we only have registrations from 2016 to 2018, further studies are needed to better clarify whether the availability of rapid response vehicles affects a PCD’s call-out to severe trauma incidents. It is possible that PCDs prefer to call out with an ambulance instead of a rapid response vehicle in cases of severe trauma incidents.

There was a substantial variation in proportions and rates of PCD call-outs to severe trauma incidents across the 16 EMCCs in Norway. There are large differences in demographics across these EMCC areas. We have documented that the call-out rates were affected by rural-urban factors. It is likely that differences in rural and urban EMCC areas could partly explain the variation in proportions and rates of call-outs. A Norwegian study from 2005 documented a significant difference, with an RR of 3.9, when comparing how often two EMCCs notified the GP in acute situations [27]. It also showed a significant difference, with an RR of 3.2, when comparing how often the GP worked within the geographical areas of these EMCCs, called out when notified. This variation was even greater in our study, with a difference in call out frequencies of more than tenfold across the EMCCs.

We can argue that the organisation of the EMCCs may have little impact on the work of the PCDs, as EMCCs and PCDs are organised in different parts of the health care system. Although PCDs work in the municipalities, they are influenced by the decisions of prehospital health personnel in the specialist health service. If the doctors are not alerted by the EMCCs about incidents with suspected severe trauma, it is not possible to call out. Future studies should investigate why there are such variations across the EMCCs.

This study is based on national registers that include all acute somatic hospital admissions from 2012 to 2018 [16]. By using this large data set, we avoided selection bias of sub-groups when we conducted our further analysis. The registries made it possible for us to investigate how variations in rural-urban settings and time factors were associated with PCD call-outs to severe trauma incidents in Norway. Previous studies have clearly demonstrated the connection between claims from emergency primary health care and hospital admissions less than 24 h later. This supports the design of this study. By using this method, our registers provide us with the number of call-outs and the total number of PCD engagements during the study period. This means that we can calculate with a high degree of certainty the percentage of call-outs when the PCDs were involved. Of 4,342 severe trauma incidents, we observed PCD involvement in 1,683 incidents. For the other 2,659 incidents (61%) we do not have any documented PCD involvement. It is highly probable that the PCDs were not involved.

According to the discharge diagnoses, these were cases of suspected severe trauma. The EMCC guidelines state that both the ambulance and PCD should be alarmed in cases of suspected severe trauma, and it would be concerning if many of these incidents were handled without any kind of alarm being sent to the PCD. A Second possibility could be that incidents involved a severe trauma with limited initial symptoms that were handled by a PCD clinic consultation or a telephone contact prior to an alarm from the EMCC to the ambulance. We recommend further studies to investigate the degree to which EMCCs send alarms to PCDs in the event of severe trauma incidents.

The registries used in our research did not include information about severely injured patients who died before admission or in the hospital. The Norwegian Cause of Death Registry reported 2.7% mortality due to trauma for the years 2012–2018, and the Norwegian Trauma Registry found 3.2% mortality 30 days after trauma for all patients admitted to Norwegian trauma hospitals for the years 2016–2018 [38, 39]. Consequently, deaths account for only a limited proportion of the overall trauma incidents. Probably, including dead trauma patients would have very small impact on the results.

At an early stage of severe trauma, the information in the primary message from the EMCC to the PCD and the ambulance is almost identical. It will be difficult to distinguish an incident where the patient dies later in the course from a patient who survives. Therefore, there is likely an equal frequency of call-outs from the PCD in both cases.

We used the patients’ home municipalities for distribution across the EMCCs. To ensure a more correct distribution, we should have used the municipalities in which the incident occurred. As we did not have access to that information, the actual distribution across the EMCCs may differ slightly from what we have reported. However, although the incident may not have happened within the patient’s own municipality, we think that it may relatively often have happened within the same geographical area of the EMCC.

Most injuries with an ICD-10 code in blocks S-T are due to minor injuries and can be handled within a lower emergency care level. It was important to ensure that we did not include minor injuries, which would not have triggered an acute alarm. We achieved this by including only significant multitrauma patients with DRG codes 484–487. It is a limitation of the study that injuries without these DRG codes were thereby excluded. A larger sample would have strengthened the statistical power of the study.

## Conclusions

Centrality affects the proportion of PCD call-outs to severe trauma incidents, and call-out rates were higher in rural than in urban areas. We found no significant difference in call-out rates according to time factors. Possible consequences of these findings should be further investigated.

## Data Availability

The data used in this study was provided by the Norwegian Directorate of Health and Statistics Norway, with restrictions to only be used under licence for researchers in the current study and so is not publicly available. However, the registry data used in this study will be available from the authors upon reasonable request and with included permission from the Norwegian Directorate of Health, Statistics Norway, the Regional Ethical Committee and the Norwegian Data Protection Authority.

## Abbreviations

PCD: Primary Care Doctor
CI: Confidence Interval
HEMS: Helicopter Emergency Medical Services
South-East: South-Eastern Norway
West: Western Norway
Central: Central Norway
North: Northern Norway
EMCC: Emergency Medical Communication Centre
KUHR: Control and Payment of Reimbursement to Health Service Providers database
NPR: Norwegian Patient Registry
NOOHR: The National Out-Of-Hours Services Registry
GP: General Practitioner
ICD-10: International Statistical Classification of Diseases and Related Health Problems, Version 10
DRG: Diagnosis-Related Group
GLM: generalised linear models
RR: Relative Risk

## References

[1] Saunes IS, Karanikolos M, Sagan A, Norway (2020). Health system review. Health Syst Transition nor Inst Public Health.

[2] McSwain N, Rotondo M, Meade P, Duchesne J (2012). A model for rural trauma care. Br J Surg.

[3] Morgan JM, Calleja P (2020). Emergency trauma care in rural and remote settings: challenges and patient outcomes. Int Emerg Nurs.

[4] Fatovich DM, Jacobs IG (2009). The relationship between remoteness and trauma deaths in Western Australia. J Trauma.

[5] Steinvik T, Raatiniemi L, Mogensen B, Steingrímsdóttir GB, Beer T, Eriksson A (2022). Epidemiology of trauma in the subarctic regions of the nordic countries. BMC Emerg Med.

[6] Cnossen MC, van der Brande R, Lingsma HF, Polinder S, Lecky F, Maas AIR (2018). Prehospital trauma care among 68 European neurotrauma centers: results of the CENTER-TBI provider profiling questionnaires. J Neurotrauma.

[7] Taran S (2009). The Scoop and Run Method of Pre-clinical Care for Trauma victims. Mcgill J Med.

[8] Spoelder EJ, Slagt C, Scheffer GJ, van Geffen GJ (2022). Transport of the patient with trauma: a narrative review. Anaesthesia.

[9] Langhelle A, Lossius HM, Silfvast T, Björnsson HM, Lippert FK, Ersson A (2004). International EMS systems: the nordic Countries. Resuscitation.

[10] Regional Health Authorities. Traumesystemet i Norge. Traumeplan NKT (Trauma care system in Norway)(In Norwegian). 2015. Accessed 15 October 2023.

[11] Committee on Trauma, American College of Surgeons. Resources for optimal care of the injured patient. Chicago, Il: American College of Surgeons. 2022. https://www.resources-for-optimal-care.pdf(facs.org). Accessed 15 October 2023.

[12] Forskrift om krav til. Og organisering av kommunal legevaktordning, ambulansetjeneste, medisinsk nødmelddetjeneste mv. (regulation on organisation of emergency services) (in Norwegian). 2015. Accessed 15 October 2023.

[13] Allertsen M, Morken T. Legevaktorganisering i Norge. Rapport fra Nasjonalt legevaktregister 2022. Rapport nr. 4-2022. Bergen: Nasjonalt kompetansesenter for legevaktmedisin, NORCE Norwegian Research Centre, (The organization of emergency primary care centres in Norway)(In Norwegian). 2022. Accessed 15 October 2023.

[14] 09556. Ambulance services. Number of ambulances, operating hours, assignments and kilometres driven, by health enterprise 2011–2021. Statbank Norway (ssb.no) 2022. https://www.ssb.no/en/statbank/table/09556/. Accessed 15 October 2023.

[15] About the National Air Ambulance Services of Norway. (Air Ambulance Serv Norway). 2022. 15 October 2023.

[16] Myklevoll KR, Zakariassen E, Morken T, Baste V, Blinkenberg J, Bondevik GT (2023). Primary care doctors in acute call-outs to severe trauma incidents in Norway – associations with factors related to patients and doctors. Scand J Prim Health Care.

[17] Blinkenberg J, Pahlavanyali S, Hetlevik Ø, Sandvik H, Hunskaar S (2019). General practitioners’ and out-of-hours doctors’ role as gatekeeper in emergency admissions to somatic hospitals in Norway: registry-based observational study. BMC Health Serv Res.

[18] Blinkenberg J, Pahlavanyali S, Hetlevik Ø, Sandvik H, Hunskaar S (2020). Correction to: general practitioners’ and out-of-hours doctors’ role as gatekeeper in emergency admissions to somatic hospitals in Norway: registry-based observational study. BMC Health Serv Res.

[19] Boland M, Staines A, Fitzpatrick P, Scallan E (2005). Urban-rural variation in mortality and hospital admission rates for unintentional injury in Ireland. Inj Prev.

[20] Zwerling C, Peek-Asa C, Whitten PS, Choi S-W, Sprince NL, Jones MP (2005). Fatal motor vehicle crashes in rural and urban areas: decomposing rates into contributing factors. Inj Prev.

[21] Fatovich DM, Phillips M, Langford SA, Jacobs IG (2011). A comparison of metropolitan vs rural major trauma in Western Australia. Resuscitation.

[22] Gabella B, Hoffman RE, Marine WW, Stallones L (1997). Urban and rural traumatic brain injuries in Colorado. Ann Epidemiol.

[23] Gomez D, Berube M, Xiong W, Ahmed N, Haas B, Schuurman N (2010). Identifying targets for potential interventions to reduce rural trauma deaths: a population-based analysis. J Trauma.

[24] Gonzalez RP, Cummings G, Mulekar M, Rodning CB (2006). Increased mortality in rural vehicular trauma: identifying contributing factors through data linkage. J Trauma.

[25] Raatiniemi L, Steinvik T, Liisanantti J, Ohtonen P, Martikainen M, Alahuhta S (2016). Fatal injuries in rural and urban areas in northern Finland: a 5-year retrospective study. Acta Anaesthesiologia Scandinavia.

[26] Hjortdahl M, Halvorsen P, Risør MB (2016). Rural GPs’ attitudes toward participating in emergency medicine: a qualitative study. Scand J Prim Health Care.

[27] Vaardal B, Lossius HM, Johnsen R (2005). Have the implementation of a new specialised emergency medical service influenced the pattern of general practitioners’ involvement in pre-hospital medical emergencies? A study of geographic variations in alerting, dispatch, and response. Emerg Med J.

[28] Helman CG (1984). The role of context in primary care. J Royal Coll Gen Practitioners.

[29] Hjortdahl M, Zakariassen E, Wisborg T (2014). The role of general practitioners in the pre hospital setting, as experienced by emergency medicine technicians: a qualitative study Scandinavia journal of Trauma. Resusc Emerg Med.

[30] Chng CL, Collins J, Eaddy S (2001). A comparison of rural and urban Emergency Medical System (EMS) personnel: a Texas study. Prehosp Disaster Med.

[31] Bentley MA, Shoben A, Levine R (2016). The Demographics and Education of Emergency Medical Services (EMS) professionals: a National Longitudinal Investigation. Prehosp Disaster Med.

[32] Zakariasssen E, Hunskaar S (2010). Involvement in emergency situations by primary care doctors on-call in Norway–a prospective population-based observational study. BMC Emerg Med.

[33] Reilly PM, Schwab CW, Branas CC, Geiger J, Wiebe DJ (2011). Weekend and night outcomes in a statewide trauma system. Arch Surg.

[34] Fitschen-Oestern S, Lippross S, Lefering R, Klüter T, Weuster M, Franke GM (2021). Does the time of the day affect multiple trauma care in hospitals? A retrospective analysis of data from the TraumaRegister DGU^®^. BMC Emerg Med.

[35] Pape-Köhler CLA, Simanski C, Nienaber U, Lefering R (2014). External factors and the incidence of severe trauma: Time, date, season and moon. Injury.

[36] Søreide k (2010). Temporal patterns of death after trauma: evaluation of circadian, diurnal, periodical and seasonal trends in 260 fatal injuries. Scand J Surg.

[37] Ovadia P, Szewczyk D, Walker K, Abdullah F, Schmidt-Gillespie S, Rabinovici R (2000). Admission patterns of an urban level I trauma center. Am J Med Qual.

[38] Norwegian Cause of Death Registry. (The official cause of death statistics for Norway) Accessed 5 June 2024.

[39] Norwegian Trauma Registry (NTR) Accessed 5 June 2024.

